# Multimorbidity and associated informal care receiving characteristics for US older adults: a latent class analysis

**DOI:** 10.1186/s12877-024-05158-z

**Published:** 2024-07-03

**Authors:** Ruotong Liu, Corey L. Nagel, Siting Chen, Jason T. Newsom, Heather G. Allore, Ana R. Quiñones

**Affiliations:** 1 Department of Family Medicine, Oregon Health & Science University, 3181 SW Sam Jackson Park Road, Portland, OR 97239 USA; 2 College of Nursing, University of Arkansas for Medical Sciences, Little Rock, Arkansas, USA; 3 OHSU-PSU School of Public Health, Portland, OR USA; 4 Department of Psychology, Portland State University, Portland, OR USA; 5 Department of Internal Medicine, Yale University, New Haven, Connecticut, USA; 6 Department of Biostatistics, Yale University, New Haven, Connecticut, USA

**Keywords:** Multiple chronic conditions, Multimorbidity, Caregiving, Latent class analysis, Chronic disease combinations

## Abstract

**Background:**

Older adults with varying patterns of multimorbidity may require distinct types of care and rely on informal caregiving to meet their care needs. This study aims to identify groups of older adults with distinct, empirically-determined multimorbidity patterns and compare characteristics of informal care received among estimated classes.

**Methods:**

Data are from the 2011 National Health and Aging Trends Study (NHATS). Ten chronic conditions were included to estimate multimorbidity patterns among 7532 individuals using latent class analysis. Multinomial logistic regression model was estimated to examine the association between sociodemographic characteristics, health status and lifestyle variables, care-receiving characteristics and latent class membership.

**Results:**

A four-class solution identified the following multimorbidity groups: some somatic conditions with moderate cognitive impairment (30%), cardiometabolic (25%), musculoskeletal (24%), and multisystem (21%). Compared with those who reported receiving no help, care recipients who received help with household activities only (OR = 1.44, 95% CI 1.05–1.98), mobility but not self-care (OR = 1.63, 95% CI 1.05–2.53), or self-care but not mobility (OR = 2.07, 95% CI 1.29–3.31) had greater likelihood of being in the multisystem group versus the some-somatic group. Having more caregivers was associated with higher odds of being in the multisystem group compared with the some-somatic group (OR = 1.09, 95% CI 1.00-1.18), whereas receiving help from paid helpers was associated with lower odds of being in the multisystem group (OR = 0.36, 95% CI 0.19–0.77).

**Conclusions:**

Results highlighted different care needs among persons with distinct combinations of multimorbidity, in particular the wide range of informal needs among older adults with multisystem multimorbidity. Policies and interventions should recognize the differential care needs associated with multimorbidity patterns to better provide person-centered care.

**Supplementary Information:**

The online version contains supplementary material available at 10.1186/s12877-024-05158-z.

## Introduction

The estimated prevalence of multimorbidity, defined as the coexistence of two or more chronic conditions [1] was 73%, or 38 million people, among adults aged 65 and older in the United States in 2019 [2]. A systematic review and meta-analysis revealed that compared with people without multimorbidity, the risk of death was 1.73 and 2.72 times higher for people with 2 or more and 3 or more morbidities, respectively [3]. In addition to higher mortality risk, multimorbidity is associated with increased healthcare utilization and expenditures, heightened risks of disability and frailty, and decreased health-related quality of life [4–6].

Given its importance, multimorbidity has been the focus of a growing body of research. However, the measures of multimorbidity employed across studies show considerable variation. Ho and colleagues (2021) [7] conducted a systematic review of 566 studies on multimorbidity and found that 66.4% studies used a count of conditions as the measure for multimorbidity, and 27.4% used weighted indices, such as the Charlson Comorbidity Index or the Cumulative Illness Rating Scale-Geriatric. While these measures are useful for descriptive purposes and helpful to gauge total burden [8], index-based measures only provide high-level summaries and little information on specific disease combinations. Another limitation noted in the review was that less than half of the studies incorporated mental health conditions, such as depression, despite their well-established collective impact on an individual’s quality of life and disability [9–12]. As a consequence, the authors strongly advocated for the inclusion of mental health conditions in the research of multimorbidity [7].

Studies that examine the specific patterns of chronic conditions can enhance our understanding of the unique needs of older adults associated with their varied and evolving multimorbidity patterns. Among studies that examine distinct patterns of multimorbidity, latent class analysis (LCA) is a valuable methodological approach [13, 14]. LCA is a person-centered method based on structural equation modeling that can be utilized to decompose the heterogeneity among samples in which classes are expected to be categorically distinct [15]. It can be helpful in identifying clusters of individuals who share similar patterns of chronic condition combinations. Several studies conducted in the United States that employed LCA methodologies to examine multimorbidity patterns have identified distinct patterns of chronic disease combinations associated with frailty [16], healthcare utilization [17], and mortality [18].

Comparatively few studies have investigated the informal care needs of older adults and how the types and levels of assistance received for those needs is differentially associated with various multimorbidity patterns. Specifically, multimorbidity is associated with reduced functional health, including limitations in activities of daily living (ADLs) and instrumental activities of daily living (IADLs), in part due to higher levels of symptom burden [12, 19–21]. Persons with different multimorbidity patterns, therefore, could require distinct levels of care in ADLs and IADLs. Unpacking care-receiving characteristics associated with multimorbidity patterns could provide greater insight into the anticipated informal care needs and supports for older adults with various disease combinations, and allow for models of care to better support these needs and the needs of their caregivers.

Therefore, the aim of the current study is twofold. First, to identify distinct multimorbidity patterns of a discrete set of self-reported physical and mental health conditions by applying LCA to a nationally-representative sample of older adults in the United States. Second, to delineate the informal care-receiving characteristics that are distinctively associated with the identified multimorbidity patterns.

## Methods

### Data

The data used were from Round 1 (collected in 2011) and Round 5 (collected in 2015) of the National Health and Aging Trends Study (NHATS). NHATS is led by the Johns Hopkins University Bloomberg School of Public Health and is sponsored by the National Institute on Aging (U01AG032947). The study contains a nationally-representative sample of Medicare beneficiaries aged 65 and older in the United States. The participants complete in-person interviews annually to collect information on disablement and its consequences [22]. NHATS is replenished periodically, and in Round 5, a new sample of beneficiaries was introduced. There were 8245 participants in 2011 and 4182 newly introduced participants in 2015 [23]. For this study, care-receiving information was obtained from NHATS other person (OP) and sample person (SP) data. After merging the SP file with OP file, a total of 7532 (91.35%) participants were included in the merged Round 1 data, and 3870 (92.54%) participants were included in the replenished Round 5 data.

### Measures

The outcome of interest is multimorbidity patterns. Models were adjusted for care-recipients’ sociodemographic characteristics, health status, lifestyle variables, and care-receiving characteristics. The selection of variables was informed by previous research examining multimorbidity patterns [24] and caregiving/care-receiving outcomes including systematic reviews and meta analyses [25, 26], and the most recent caregiving research [27].

**Multimorbidity patterns**. Care recipient relative probability of having each of the ten included chronic conditions was used to identify latent classes (groups) of persons with similar patterns of multimorbidity. Respondents (or their proxies) were asked if a physician had previously diagnosed any of the conditions, including cardiac condition, high blood pressure, arthritis, osteoporosis, diabetes, lung disease, stroke, and cancer. A yes (1) or no (0) response indicated the presence of any of the 10 condition(s) at each interview. Depressive symptoms were measured using the Patient Health Questionnaire 2 (PHQ-2), a validated screening instrument for depression in older adults [28], with a PHQ-2 score of 3 and above indicating a positive screen for depression [29]. Dementia status was determined following the validated NHATS classification including probable dementia, possible dementia, and no dementia [30]. The NHATS definition shows good sensitivity and specificity of the measure when validated against the Aging, Demographics, and Memory Study (ADAMS) [31]. Participants with possible or probable dementia were categorized as having cognitive impairment in the current study.

**Sociodemographic variables**. Care recipient age was measured in years at the time of the interview. Sex included male and female with male as the reference group. Race/ethnicity was classified into four categories: non-Hispanic White (reference group), non-Hispanic Black, Hispanic, and Other (including American Indian, Asian, and native Hawaiian). Education ranged from 1 “no schooling completed” to 9 “master’s, professional, or doctoral”, which were then grouped into “high school and below” (reference group), “some college”, or “college and above”. Marital status was classified into four-categories indicating “married/partnered” (reference group), “separated/divorced”, “widowed”, and “never married”. Whether the participant was born in the United States, received Medicaid, lived in community (0) or residential care that are not nursing homes (1), and proxy respondent status were also included. ADLs (7 items: dressing, eating, bathing, toileting, transferring from bed, getting around inside, and going outside) and IADLs (5 items: cleaning laundry, preparing hot meals, grocery shopping, taking medications, and managing money), were each summed and included as count variables. Body mass index (BMI) was calculated according to the established formula (BMI = weight [pounds] x 703 / height^2^[inches]), and was classified into four categories: underweight (BMI < 18.5), healthy weight (BMI = 18.5 to < 25.0), overweight (BMI = 25 to < 30.0), and obese (BMI ≥ 30). Whether the participant had ever smoked cigarettes was included as a binary variable. Self-rated health was a continuous variable ranging from “1-poor” to “5-excellent” health.

**Care-receiving characteristics**. The numbers of persons providing care to the care recipients were included. The NHATS asks whether assistance was received for health or functioning reasons for household activities. We utilized this item to create a five-hierarchy categorical variable following the classification by Wolff and Spillman (2014). This variable included five mutually exclusive categories: “no assistance received for health or functioning reasons”, “household activities only, for health and functioning reasons”, “mobility but not self-care activities”, “self-care but not mobility activities” and “both mobility and self-care activities” with the last three categories constructed regardless of the help received for household activities. We also included a variable indicating whether the participant received help from paid helpers (yes = 1, no = 0).

### Analysis

Data analysis was conducted in four steps. First, latent class analysis (LCA) was applied to identify distinguishable multimorbidity classes. The advantages of LCA include that it is not constrained by the prior specification of group distributions [32] or the requirement of continuous indicators [33]. LCA identifies homogenous groups by computing the posterior class probabilities and probabilities of item response conditional on class membership [34]. Mutually exclusive and collectively exhaustive latent classes are identified so that the homogeneity within and heterogeneity across classes are maximized. For the current study, latent classes were estimated starting from one-class solutions until convergence could not be achieved. The optimal number of classes was decided by considering multiple factors, including the Bayesian Information Criterion [35], entropy, and Bootstrap Likelihood Ratio Test (BLRT). Lower BIC values indicated a better fit of the model [36], higher entropy indicated lower classification error and a more precise classification [37], and the BLRT compared models to decide whether the K-class model outperformed the (K-1)-class model [38, 39]. Parsimony and interpretability of results were also considered in model selection. LCA calculates individual’s probability of class membership in each latent class and each participant was assigned to the class with the highest posterior probability of membership, which was used in the subsequent multinomial logistic regression models [37].

Sociodemographic characteristics, health status and lifestyle variables, and care-receiving characteristics were described in the second step for both the whole sample and by identified classes. The percentages of individuals who received assistance with various household activities (laundry, shopping, meal preparation, and managing bills and banking), self-care activities (eating, dressing, bathing, toileting), and mobility activities (moving around indoors and outdoors and transferring from bed), were calculated to describe the care-receiving attributes of the participants. Third, a multinomial logistic regression model was estimated to examine the association between included variables and group membership. We utilized full covariate adjustment for sociodemographic characteristics, health status and lifestyle variables, and care-receiving characteristics. In order to account for the inherent uncertainty in latent class assignment, participants were weighted using the posterior probability of assigned group membership [40, 41]. At last, the same LCA analyses were performed on both the replenished cohort from Round 5 (Appendix Table 1) and among self-respondents from Round 1 (Appendix Table 2) to examine the replicability and stability of identified classes. The calculation of BLRT was conducted using Mplus 8.4., and all the remaining analyses were conducted using Stata/SE 17.0.

## Results

### Latent class analysis results

#### Model selection

After calculating fit indices, BIC and entropy were in favor of a four- or five-class model, but the BLRT was in favor of a five-class model. Taking model interpretability into consideration, the distribution of participants was more balanced in the four-class model whereas the five-class model generated two groups with relatively small portions of observations (12–17%). Therefore, the four-class model was selected in the final analysis. In the final four-class model, the BIC is 78962.65, BLRT is 242.02, and entropy is 0.399.

### Multimorbidity patterns

Table 1 displays the four groups based on the probability of occurrence of the ten included chronic conditions. The four latent classes are (1) some somatic conditions with moderate cognitive impairment group (hereafter “some-somatic” group) (30%), characterized by low probability of conditions except for possible or probable dementia; (2) cardiometabolic group (25%) with higher factor loadings in high blood pressure (0.96) and diabetes (0.40), and lower loadings in osteoporosis (0.04) compared to the other groups; (3) musculoskeletal and other somatic group (23%), characterized by high loadings in arthritis (0.78), osteoporosis (0.42), cancer (0.33) but low loadings in dementia (0.10) and depression (0.07); and (4) multisystem group (22%) with high loadings in all ten conditions, across domains.

**Table 1:** Four class solution: prevalence of latent classes and factor loadings (*n* = 7532)

	Class 1 30% *n* = 2260 Some-Somatic	Class 2 25% *n* = 1883 Cardiometabolic	Class 3 23% *n* = 1732 Musculoskeletal	Class 4 22% *n* = 1657 Multisystem
Cardiac condition	0.10	0.31	0.20	0.50
High blood pressure	0.33	0.96	0.67	0.81
Arthritis	0.28	0.45	0.78	0.82
Osteoporosis	0.08	0.04	0.42	0.33
Diabetes	0.10	0.40	0.12	0.42
Lung disease	0.06	0.11	0.21	0.26
Stroke	0.04	0.11	0.04	0.31
Cancer	0.18	0.27	0.33	0.26
Dementia	0.25	0.20	0.10	0.53
Depression	0.08	0.08	0.07	0.45

Note: Some-Somatic refers to the some-somatic with moderate cognitive impairment group; dementia includes possible and probable dementia classifications assessed in NHATS

#### Profiles of multimorbidity classes

Table 2 displays characteristics for the whole sample and for each identified group. For sociodemographic characteristics, the some-somatic group was the youngest (76.82, SD = 8.14), followed by the musculoskeletal (77.09, SD = 7.45), cardiometabolic (77.41, SD = 7.46), and multisystem (79.91, SD = 8.05) groups. About half the participants were female in the some-somatic (49.87%) and cardiometabolic (48.58%) groups; however, the proportion of female was higher in the multisystem and musculoskeletal groups, with almost two-thirds (63.73%) and three-fourths (74.31%) female, respectively. There were more non-Hispanic White participants in the some-somatic and musculoskeletal groups, non-Hispanic Black participants were more likely to be assigned to the cardiometabolic and multisystem groups, and Hispanics participants were more likely to be assigned to the multisystem group. The some-somatic group had more education and higher proportions of people married or partnered, whereas the multisystem group had the lowest level of educational attainment and a higher proportion of people who are widowed. Relatively more people were born in a foreign country in both the some-somatic and multisystem groups compared to the other two groups. Moreover, the proportions of people receiving Medicaid were the highest in the multisystem group (28.57%) and the lowest in the musculoskeletal group (9.92%), and a higher proportion (7.78%) of care recipients in the multisystem group lived in residential care.

For health status and lifestyle variables, the multisystem group had the highest number of functional limitations, including ADLs and IADLs, followed by the cardiometabolic, musculoskeletal, and some-somatic groups. Using BMI, people categorized as obese were overrepresented in the cardiometabolic group, whereas the multisystem group had the highest proportions of people categorized as underweight. Self-rated health was the highest among the some-somatic group, followed by the musculoskeletal, cardiometabolic, and multisystem groups.

For care-receiving characteristics, a larger proportion of participants in the multisystem group required 2 or more caregivers compared to those in the other three groups. As anticipated, the largest proportion of people receiving help in household, self-care activities, and mobility activities fell in the multisystem group, who has the highest intensity of functional limitations. The smallest proportion of people receiving help in both self-care and mobility activities fell in the musculoskeletal group. In addition, a higher proportion (2.06%) of care recipients in the multisystem group received help from paid helpers. The proportion of participants receiving help in each specific help domain and ADL/IADL items can be found in Table 3 in Appendix.

**Table 2:** Sample characteristics

	All	Some-Somatic	Cardiometabolic	Musculoskeletal	Multisystem
	*n* = 7532	*n* = 2292 (30%)	*n* = 1871 (25%)	*n* = 1814 (24%)	*n* = 1555 (21%)
**Sociodemographic**					
Age	77.67 (7.88)	76.82 (8.14)	77.41 (7.46)	77.09 (7.45)	79.91 (8.05)
Female	4391 (58.30%)	1143 (49.87%)	909 (48.58%)	1348 (74.31%)	991 (63.73%)
Race/Ethnicity - Non-Hispanic White - Non-Hispanic Black - Hispanic - Others	5134 (68.89%) 1649 (22.13%) 451 (6.05%) 218 (2.93%)	1632 (72.15%) 398 (17.60%) 153 (6.76%) 79 (3.49%)	1178 (63.47%) 532 (28.66%) 95 (5.12%) 51 (2.75%)	1410 (78.20%) 282 (15.64%) 73 (4.05%) 38 (2.11%)	914 (59.70%) 437 (28.54%) 130 (8.49%) 50 (3.27%)
Education - High school and below - Some college - College and above	4073 (54.74%) 1802 (24.22%) 1566 (21.05%)	1121 (49.58%) 543 (24.02%) 597 (26.40%)	1019 (54.99%) 447 (24.12%) 387 (20.89%)	868 (48.14%) 511 (28.34%) 424 (23.52%)	1065 (69.88%) 301 (19.75%) 158 (10.37%)
Marital status - Married/partnered - Separated/divorced - Widowed - Never married	3770 (50.11%) 915 (12.16%) 2543 (33.80%) 296 (3.93%)	1286 (56.21%) 273 (11.93%) 619 (27.05%) 110 (4.81%)	1006 (53.83%) 217 (11.61%) 584 (31.25%) 62 (3.32%)	878 (48.45%) 225 (12.41%) 651 (35.93%) 58 (3.20%)	600 (38.59%) 200 (12.86%) 689 (44.31%) 66 (4.24%)
Foreign born	840 (11.26%)	308 (13.59%)	170 (9.16%)	136 (7.54%)	226 (14.72%)
Medicaid	1159 (15.81%)	292 (13.17%)	260 (14.24%)	177 (9.92%)	430 (28.57%)
Live in residential care	399 (5.30%)	112 (4.89%)	88 (4.70%)	78 (4.30%)	121 (7.78%)
Proxy respondents	571 (7.58%)	140 (6.11%)	93 (4.97%)	46 (2.54%)	292 (18.78%)
**Health status and lifestyle**					
ADL − 0 − 1 − 2 to 3 − 4 to 7	4377 (58.13%) 1053 (13.98%) 991 (13.17%) 1109 (14.73%)	1713 (74.74%) 255 (11.13%) 169 (7.38%) 155 (6.76%)	1152 (61.60%) 296 (15.83%) 232 (12.40%) 190 (7.17%)	1112 (61.30%) 287 (15.82%) 242 (13.34%) 173 (9.64%)	400 (25.74%) 215 (13.84%) 248 (22.40%) 591 (38.03%)
IADL − 0 − 1 − 2 to 3 − 4 to 5	4530 (60.15%) 1024 (13.60%) 1022 (13.57%) 955 (12.68%)	1723 (75.17%) 231 (10.08%) 183 (7.98%) 155 (6.76%)	1191 (63.69%) 296 (15.83%) 235 (12.57%) 148 (7.92%)	1173 (64.66%) 277 (15.27%) 243 (13.40%) 121 (6.67%)	443 (28.49%) 220 (14.15%) 361 (23.22%) 531 (34.15%)
BMI - Underweight - Normal - Overweight - Obese	190 (2.52%) 2435 (32.33%) 2655 (35.25%) 2252 (29.90%)	68 (2.97%) 880 (38.39%) 842 (36.74%) 502 (21.90%)	27 (1.44%) 472 (25.23%) 685 (36.61%) 687 (36.72%)	33 (1.82%) 627 (34.56%) 634 (34.95%) 520 (28.67%)	62 (3.99%) 456 (29.32%) 494 (31.77%) 543 (34.92%)
Smoking (ever)	3795 (50.45%)	1124 (49.06%)	987 (52.87%)	909 (50.11%)	775 (49.97%)
Self-rated health	3.15 (1.13)	3.72 (1.03)	3.04 (0.98)	3.24 (1.01)	2.33 (1.04)
**Care-receiving characteristics**					
Number of helpers − 0 − 1 − 2 − 3 and above	1141 (15.21%) 3322 (44.29%) 1792 (23.89%) 1246 (16.61%)	435 (19.07%) 1147 (50.28%) 437 (19.16%) 262 (11.49%)	262 (14.04%) 874 (46.84%) 461 (24.71%) 269 (14.42%)	324 (17.97%) 802 (44.48%) 419 (23.24%) 258 (14.31%)	120 (7.74%) 499 (32.17%) 475 (30.63%) 457 (29.45%)
Care-receiving categories - Received no assistance - Household activities only - Mobility but not self-care - Self-care but not mobility - Both self-care and mobility	5331 (70.78%) 997 (13.24%) 389 (5.16%) 281 (3.73%) 534 (7.09%)	1902 (82.98%) 192 (8.38%) 76 (3.32%) 48 (2.09%) 74 (3.23%)	1415 (75.63%) 208 (11.12%) 85 (4.54%) 68 (3.63%) 95 (5.08%)	1412 (77.84%) 225 (12.40%) 71 (3.91%) 51 (2.81%) 55 (3.03%)	602 (38.71%) 372 (23.92%) 157 (10.10%) 114 (7.33%) 310 (19.94%)
Receive help from paid helper	88 (1.17%)	22 (0.96%)	26 (1.39%)	8 (0.44%)	32 (2.06%)

Note: mean (SD) included for continuous variables; n (%) included for categorical variables; Some-Somatic refers to the some-somatic with moderate cognitive impairment group

### Factors associated with multimorbidity patterns

Table 3 displays the multinomial logistic regression results. The some-somatic group served as the reference group. Participants who were older (odds ratio [OR] = 1.02, 95% CI 1.00-1.03), non-Hispanic Black (OR = 1.61, 95% CI 1.35–1.92), Medicaid recipients (OR = 1.28, 95% CI 1.03–1.60), overweight (OR = 1.63, 95% CI 1.38–1.92), or obese (OR = 2.74, 95% CI 2.28–3.29) were more likely to be in cardiometabolic group relative to the some-somatic group, whereas participants who were never married (OR = 0.57, 95% CI 0.39–0.84), born in a foreign country (OR = 0.61, 95% CI 0.47–0.80), underweight (OR = 0.47, 95% CI 0.29–0.77), or had higher self-rated health (OR = 0.50, 95% CI 0.46–0.54) were less likely to be classified in the cardiometabolic group compared to the some-somatic group.

For the comparison between the musculoskeletal and the some-somatic group, female sex (OR = 4.20, 95% CI 3.57–4.93), having some college (OR = 1.26, 95% CI 1.06–1.49) or above-college education (OR = 1.47, 95% CI 1.23–1.76), being a Medicaid recipient (OR = 1.42, 95% CI 1.12–1.80), having more ADL limitations (OR = 1.15, 95% CI 1.06–1.25), being obese (OR = 1.34, 95% CI 1.11–1.61), or being a smoker (OR = 1.28, 95% CI 1.11–1.48) were risk factors for being in the musculoskeletal group. Participants who were non-Hispanic Black (OR = 0.66, 95% CI 0.54–0.80) or Hispanic (OR = 0.56, 95% CI 0.39–0.80), never married (OR = 0.55, 95% CI 0.42–0.72), born in a foreign country (OR = 0.55, 95% CI 0.42–0.72), living in residential care (OR = 0.68, 95% CI 0.48–0.96), proxy respondents (OR = 0.42, 95% CI 0.28–0.64), underweight (OR = 0.39, 95% CI 0.24–0.64), and had better self-rated health (OR = 0.54, 95% CI 0.50–0.58) were more likely to be in the some-somatic versus musculoskeletal group.

At last, being older (OR = 1.02, 95% CI 1.01–1.03), female (OR = 1.44, 95% CI 1.19–1.73), widowed (OR = 1.29, 95% CI 1.04–1.60), with more functional limitations (ADL: OR = 1.27, 95% CI 1.17–1.37; IADL: OR = 1.20, 95% CI 1.09–1.31), overweight (OR = 1.59, 95% CI 1.30–1.94) or obese (OR = 2.23, 95% CI 1.79–2.78), a smoker (OR = 1.26, 95% CI 1.06–1.50), having more helpers (OR = 1.09, 95% CI 1.00-1.18), receiving help in household activities only (OR = 1.44, 95% CI 1.05–1.98), receiving help in mobility but not self-care (OR = 1.63, 95% CI 1.05–2.53), or receiving help in self-care activities but not mobility (OR = 2.07, 95% CI 1.29–3.31) were risk factors for being in the multisystem group. Participants who were Hispanic (OR = 0.58, 95% CI 0.40–0.85), had a college education or above (OR = 0.69, 95% CI 0.54–0.88), underweight (OR = 0.52, 95% CI 0.32–0.85), had better self-rated health (OR = 0.34, 95% CI 0.31–0.37), or received help from paid helpers (OR = 0.36, 95% CI 0.16–0.77) were associated with lower odds of falling in the multisystem group relative to the some-somatic ﻿group.

**﻿Table 3:** Multinomial regression analysis for sociodemographic and health indicators associated with latent classes (*n* = 7532)

	Class 2		Class 3		Class 4	
	*Cardiometabolic*		*Musculoskeletal*		*Multisystem*	
	OR(SE)	95% CI	OR (SE)	95% CI	OR (SE)	95% CI
Age	1.02	[1.00, 1.03]	1.00	[0.99, 1.01]	1.02	[1.01, 1.03]
Female	0.97	[0.83, 1.13]	4.20	[3.57, 4.93]	1.44	[1.19, 1.73]
Race/ethnicity (ref: NH White) - NH Black - Hispanic - Other	1.61 0.74 1.26	[1.35, 1.92] [0.52, 1.05] [0.83, 1.90]	0.66 0.56 0.83	[0.54, 0.80] [0.39, 0.80] [0.53, 1.29]	1.03 0.58 0.96	[0.83, 1.27] [0.40, 0.85] [0.57, 1.60]
Education (ref: <=high school) - Some college - College and above	1.17 1.11	[0.99, 1.29] [0.93, 1.32]	1.26 1.47	[1.06, 1.49] [1.23, 1.76]	0.93 0.69	[0.76, 1.14] [0.54, 0.88]
Marital status (ref: married/partnered) - Separated/divorced - Widowed - Never married	0.89 1.07 0.57	[0.71, 1.12] [0.89, 1.28] [0.39, 0.84]	0.96 1.06 0.69	[0.77, 1.20] [0.88, 1.27] [0.48, 1.01]	1.26 1.29 0.73	[0.96, 1.64] [1.04, 1.60] [0.45, 1.17]
Foreign born	0.61	[0.47, 0.80]	0.55	[0.42, 0.72]	0.82	[0.61, 1.12]
Medicaid	1.28	[1.03, 1.60]	1.42	[1.12, 1.80]	0.90	[0.71, 1.15]
Live in residential care	0.88	[0.63, 1.23]	0.68	[0.48, 0.96]	1.08	[0.75, 1.57]
Proxy respondents	1.02	[0.94, 1.10]	0.42	[0.28, 0.64]	0.97	[0.68, 1.39]
ADL	0.98	[0.89, 1.07]	1.15	[1.06, 1.25]	1.27	[1.17, 1.37]
IADL	0.98	[0.89, 1.07]	0.97	[0.89, 1.06]	1.20	[1.09, 1.31]
BMI (ref: normal) - Underweight - Overweight - Obese	0.47 1.63 2.74	[0.29, 0.77] [1.38, 1.92] [2.28, 3.29]	0.39 1.15 1.34	[0.24, 0.64] [0.97, 1.35] [1.11, 1.61]	0.52 1.59 2.23	[0.32, 0.85] [1.30, 1.94] [1.79, 2.78]
Ever smoke	1.11	[0.96, 1.28]	1.28	[1.11, 1.48]	1.26	[1.06, 1.50]
Self-rated health	0.50	[0.46, 0.54]	0.54	[0.50, 0.58]	0.34	[0.31, 0.37]
Number of helpers	1.06	[0.99, 1.14]	1.04	[0.96, 1.11]	1.09	[1.00, 1.18]
Care-receiving categories (ref: no help) - Household activities only - Mobility but not self-care - Self-care but not mobility - Both self-care and mobility	0.96 1.32 1.23 1.20	[0.71, 1.30] [0.86, 2.01] [0.77, 1.95] [0.72, 1.99]	1.06 0.98 0.99 0.69	[0.79, 1.42] [0.63, 1.53] [0.61, 1.59] [0.41, 1.18]	1.44 1.63 2.07 1.42	[1.05, 1.98] [1.05, 2.53] [1.29, 3.31] [0.86, 2.32]
Received help from paid helper	1.14	[0.55, 2.36]	0.42	[0.16, 1.08]	0.36	[0.16, 0.77]

Note: OR = odds ratio; NH = Non-Hispanic; ADL = activities of daily living; IADL = instrumental activities of daily living; BMI = body mass index; reference group: some-somatic with moderate cognitive impairment group

### Replicability of classes

To examine the stability of results across cohorts in the NHATS, we conducted a sensitivity analysis applying the same procedure on the replenished cohort in Round 5 (*n* = 3870) with newly age-eligible survey participants and among self-respondents (*n* = 6961) in Round 1. Although the size of each identified class was nominally different from that in the Round 1 cohort with all care recipients, a four-class solution with similar response probabilities across latent classes was selected based on the same set of selection criteria, which largely corroborated the identified multimorbidity combination groups (Appendix Tables 1 and 2).

## Discussion

Having acknowledged the complexity of multimorbidity and related informal care needs, the current study used LCA to identify four distinct multimorbidity combination classes and investigated the associated informal care-receiving characteristics in each of these distinct groups. Specifically, we utilized a nationally representative sample of older adults aged 65 and older who are Medicare beneficiaries, included depressive symptoms and cognitive functioning in addition to somatic conditions, and corroborated the results among the refresh cohort and among self-respondents. A pivotal progression in transitioning from exploratory inquiries to confirmatory investigations in the study of multimorbidity patterns entails the identification of replicable multimorbidity groups [42]. In the systematic review by Busija and colleagues (2019), results revealed that the two most replicable multimorbidity patterns were mental health conditions and cardio-metabolic conditions [42]. Similarly, in the current study, two out of the four latent classes – the some-somatic group and the cardiometabolic group – aligned with these patterns. Sensitivity analyses further corroborated these findings. Furthermore, while the some-somatic group was characterized by low probability of physical chronic conditions, this group had one of the highest response probabilities for possible or probable dementia.

A salient aspect of the current study was the examination of care-receiving characteristics in each of the distinct multimorbidity groups. The results further highlighted the wide range of care needs among older adults with multisystem multimorbidity. Moreover, persons who received care in household activities but not mobility or self-care activities, mobility but not self-care activities, or self-care activities but not mobility activities, were more likely to be in the multisystem group. Receiving help in household activities such as laundry, shopping, meal preparation and banking was frequently observed among older adults. Nevertheless, if an individual required care in these tasks for health and functioning reasons, even in the absence of significant need for care in ADLs, it might indicate an elevated likelihood of experiencing multisystem multimorbidity. In addition, the musculoskeletal group received the least amount of care across multiple domains. This could either suggest unmet and unaddressed care needs within this group or potentially reflect more asymptomatic conditions such as arthritis for persons within this group. Since the current data do not allow us to explore the severity of conditions, these results warrant further exploration in future studies. The current study also indicated that having a larger number of caregivers was associated with increased odds of care recipients being in the multisystem group rather than the some-somatic group. In contrast, receiving help from paid caregivers correlated with lower odds of being in the multisystem group. These findings could enhance our understanding of caregiving complexity, particularly in terms of quantity and paid status of caregivers.

Multimorbidity patterns are an emerging topic that is receiving increasing attention. The identified multimorbidity latent classes are largely consistent with existing systematic reviews Busija and colleagues (2019) reported the two most replicable multimorbidity profiles were mental health conditions and cardio-metabolic conditions [42], and Prados-Torres and colleagues (2014) reported the three most stable patterns are cardiovascular and metabolic, musculoskeletal, and mental health conditions [43]. Several other studies conducted in the United States have employed LCA to unpack multimorbidity patterns, and the latent classes were similar to those in the current investigation highlighting cardiovascular, metabolic, musculoskeletal, and mental or neuropsychiatric conditions [16–18, 44]. Our study contributes to this evolving literature by providing further evidence. The differences in this investigation’s identified multimorbidity patterns could be partially attributable to different sample characteristics and the composition of included chronic conditions.

Our study was among the first to associate informal care-receiving characteristics in ADL/IADL items with multimorbidity patterns. Previous research indicated that different multimorbidity combinations were associated with differential risks of disability, measured by a combined ADL-IADL index [12]. Other studies also reported that both ADL and IADL were crucial intermediary factors on the pathway between multimorbidity and quality of life [45, 46], and IADL was a key indicator in assessing the autonomy of community-dwelling older adults [35]. The current study explored the different care-receiving characteristics in ADL/IADL items associated with multimorbidity patterns, and could serve as a bridge between multimorbidity patterns and quality of life, for both care-recipients and caregivers.

The four multimorbidity classes identified in the current study, characterized by different groupings of mental-somatic conditions, suggests that all older adults could potentially face some challenges living with and managing multimorbidity. Results highlighted the multidomain care needs among the multisystem group, the moderate amount of care needs among the some-somatic group, and the relative low care received among the musculoskeletal group that could either be a proxy of low care needs, or a signal of unmet care needs. The lower probability of having chronic conditions but relatively higher probability of having possible or probable dementia, despite being the youngest, might suggest that this group may have a higher proportion of individuals with early or earlier onset of dementia. Future research could further discern the different care needs, unmet needs, and care-receiving characteristics among older adults with different multimorbidity patterns. Healthcare policies and practice should tailor their approaches to address the unique needs accordingly to provide adequate and specific support that could potentially improve the quality of life for both care-recipients and their caregivers.

It is important to acknowledge some limitations in our study. First, the report of somatic chronic conditions was self-reported and may be subject to recall bias and underreporting. However, previous studies have shown adequate concordance between self-reported conditions and clinical data and have acknowledged the importance of documenting self-reported outcomes of disease status [44]. In addition, while our measures of care-receiving characteristics captured the actual care received, it might not accurately represent the true care needs, especially if there are unmet needs. This discrepancy could be particularly pronounced among the multisystem multimorbidity group, which might have disproportionately high unmet needs. Moreover, the help received in the three categories – household activities, self-care activities, and mobility activities, could be moderated by the recipient’s marital status, and availability of helpers. Future research could further explore the relationship between multimorbidity patterns, care network, caregiver characteristics and care-receiving needs. In addition, while our dataset permits us to identify distinct multimorbidity patterns, it does not extend to measuring their severity, which could be correlated with the types and degrees of help received. Future studies could further explore the severity of health conditions and associated care-receiving characteristics if more granular health data is available. Finally, while over 85% of respondents reported having at least one caregiver, 70-80% stated they received no assistance, except in the multisystem groups. This discrepancy arises because we only considered care received for “health and functional reasons”. Due to the data structure, we could not differentiate between caregivers providing care for strictly “health and functional reasons” and those for other non-health or functional reasons. Similarly, the data structure prohibited us from distinguishing between hours of help received solely for health and functional reasons and those received for other non-health or functional purposes. Consequently, analyzing caregiving or care-receiving hours as an indicator of the intensity of care was not feasible.

Despite these limitations, the findings shed light on the relationship between multimorbidity patterns and informal care receiving characteristics among older adults. The insights gained from this study underscore the pressing need for a more holistic approach in healthcare, one that not only addresses medical conditions but also the informal care needs of older adults.

## Conclusion

The current study used LCA to identify four distinct multimorbidity classes with different care-receiving characteristics. Results highlight different care needs among persons with distinct combinations of multimorbidity, in particular the wide range of care needs among older adults with multisystem multimorbidity. Future studies should further investigate care needs and unmet needs among persons with different multimorbidity patterns. Policies and interventions should recognize the differential care needs associated with distinct multimorbidity patterns to better provide person- and family-centered care.

### Electronic supplementary material

Below is the link to the electronic supplementary material.

Supplementary Material 1

## Data Availability

The datasets generated and/or analyzed during the current study are available in the NHATS repository https://nhats.org/researcher/data-access.

## Appendix

**Table 1:** Four class solution: prevalence of latent classes and factor loadings on replenished cohort

	Class 1 37% n = 1,428 Some somatic	Class 2 10% n = 389 Musculoskeletal	Class 3 38% n = 1,470 Cardiometabolic	Class 4 15% n = 583 Multisystem
Cardiac condition	0.10	0.21	0.25	0.52
High blood pressure	0.35	0.70	0.89	0.89
Arthritis	0.34	0.94	0.52	0.73
Osteoporosis	0.13	0.67	0.03	0.22
Diabetes	0.06	0.16	0.45	0.45
Lung disease	0.08	0.25	0.12	0.28
Stroke	0.03	0.02	0.07	0.31
Cancer	0.21	0.30	0.25	0.26
Dementia	0.18	0.14	0.13	0.47
Depression	0.07	0.18	0.07	0.40

Note: some somatic refers to the some somatic with moderate cognitive impairment group; dementia refers to possible and probable dementia

**Table 2:** Four class solution: prevalence of latent classes and factor loadings among self-respondents

	Class 1 22% n = 1,531 Some somatic	Class 2 34% n = 2,367 Cardiometabolic	Class 3 28% n = 1,949 Musculoskeletal	Class 4 16% n = 1,114 Multisystem
Cardiac condition	0.08	0.31	0.18	0.53
High blood pressure	0.27	0.86	0.66	0.84
Arthritis	0.25	0.44	0.73	0.90
Osteoporosis	0.07	0.05	0.38	0.38
Diabetes	0.08	0.39	0.10	0.48
Lung disease	0.05	0.10	0.19	0.34
Stroke	0.02	0.12	0.05	0.28
Cancer	0.16	0.27	0.32	0.26
Dementia	0.22	0.24	0.10	0.39
Depression	0.07	0.11	0.07	0.42

Note: some somatic refers to the some somatic with moderate cognitive impairment group; dementia refers to possible and probable dementia

**Table 3:** Care-receiving characteristics for ADL/IADL items

	All	Some somatic	Cardiometabolic	Musculoskeletal	Multisystem
	n = 7532	n = 2292 (30%)	n = 1871 (25%)	n = 1814 (24%)	n = 1555 (21%)
**Household activities**					
Laundry	1,059 (14.06%)	171 (7.46%)	187 (9.99%)	154 (8.49%)	547 (35.18%)
Shopping	1,613 (21.42%)	274 (11.95%)	315 (16.84%)	274 (15.10%)	750 (48.23%)
Meals	1,079 (14.33%)	192 (8.38%)	182 (9.73%)	150 (8.27%)	555 (35.69%)
Bills and banking	1,139 (84.88%)	223 (9.73%)	200 (10.69%)	134 (7.39%)	582 (37.43%)
**Self-care activities**					
Eating	316 (4.20%)	50 (2.18%)	52 (2.78%)	24 (1.32%)	190 (12.22%)
Dressing	632 (8.39%)	90 (3.93%)	128 (6.84%)	82 (4.52%)	332 (21.35%)
Bathing	387 (5.14%)	66 (2.88%)	68 (3.63%)	36 (1.98%)	217 (13.95%)
Toileting	221 (2.93%)	35 (1.53%)	33 (1.76%)	14 (0.77%)	139 (8.94%)
**Mobility activities**					
Indoor mobility	492 (6.53%)	71 (3.10%)	90 (4.81%)	50 (2.76%)	281 (18.07%)
Outdoor mobility	703 (9.33%)	113 (4.93%)	147 (7.86%)	103 (5.68%)	340 (21.86%)
Transferring from bed	361 (4.79%)	53 (2.31%)	59 (3.15%)	31 (1.71%)	218 (14.02%)

Note: some somatic refers to the some somatic with moderate cognitive impairment group
