# Supplementary material for: Multimorbidity and associated informal care receiving characteristics for US older adults: a latent class analysis
Source: BMC Geriatr. 2024 Jul 3;24:571. doi: 10.1186/s12877-024-05158-z (PMC11221032; doi:10.1186/s12877-024-05158-z)
Supplement: Supplementary file 1 — Supplementary Material 1 [file 12877_2024_5158_MOESM1_ESM.docx]

Multimorbidity and associated care-receiving characteristics for US older adults: a latent class analysis

Appendix

Table 1. Four class solution: prevalence of latent classes and factor loadings on replenished cohort (n=3870)

|  | Class 1  37%  n=1428  Some-Somatic | Class 2  10%  n=389  Musculoskeletal | Class 3  38%  n=1470  Cardiometabolic | Class 4  15%  n=583  Multisystem |
| --- | --- | --- | --- | --- |
| Cardiac condition | 0.10 | 0.21 | 0.25 | 0.52 |
| High blood pressure | 0.35 | 0.70 | 0.89 | 0.89 |
| Arthritis | 0.34 | 0.94 | 0.52 | 0.73 |
| Osteoporosis | 0.13 | 0.67 | 0.03 | 0.22 |
| Diabetes | 0.06 | 0.16 | 0.45 | 0.45 |
| Lung disease | 0.08 | 0.25 | 0.12 | 0.28 |
| Stroke | 0.03 | 0.02 | 0.07 | 0.31 |
| Cancer | 0.21 | 0.30 | 0.25 | 0.26 |
| Dementia | 0.18 | 0.14 | 0.13 | 0.47 |
| Depression | 0.07 | 0.18 | 0.07 | 0.40 |

Note: Some-Somatic refers to the some-somatic with moderate cognitive impairment group; dementia includes possible and probable dementia classifications assessed in NHATS

Table 2. Four class solution: prevalence of latent classes and factor loadings among self-respondents (n=6961)

|  | Class 1  22%  n=1531  Some-Somatic | Class 2  34%  n=2367  Cardiometabolic | Class 3  28%  n=1949  Musculoskeletal | Class 4  16%  n=1114  Multisystem |
| --- | --- | --- | --- | --- |
| Cardiac condition | 0.08 | 0.31 | 0.18 | 0.53 |
| High blood pressure | 0.27 | 0.86 | 0.66 | 0.84 |
| Arthritis | 0.25 | 0.44 | 0.73 | 0.90 |
| Osteoporosis | 0.07 | 0.05 | 0.38 | 0.38 |
| Diabetes | 0.08 | 0.39 | 0.10 | 0.48 |
| Lung disease | 0.05 | 0.10 | 0.19 | 0.34 |
| Stroke | 0.02 | 0.12 | 0.05 | 0.28 |
| Cancer | 0.16 | 0.27 | 0.32 | 0.26 |
| Dementia | 0.22 | 0.24 | 0.10 | 0.39 |
| Depression | 0.07 | 0.11 | 0.07 | 0.42 |

Note: Some-Somatic refers to the some-somatic with moderate cognitive impairment group; dementia includes possible and probable dementia classifications assessed in NHATS

Table 3. Care-receiving characteristics for ADL/IADL items

|  | All | Some-Somatic | Cardiometabolic | Musculoskeletal | Multisystem |
| --- | --- | --- | --- | --- | --- |
|  | n=7532 | n=2292 (30%) | n=1871 (25%) | n=1814 (24%) | n=1555 (21%) |
| **Household activities** |  |  |  |  |  |
| Laundry | 1059 (14.06%) | 171 (7.46%) | 187 (9.99%) | 154 (8.49%) | 547 (35.18%) |
| Shopping | 1613 (21.42%) | 274 (11.95%) | 315 (16.84%) | 274 (15.10%) | 750 (48.23%) |
| Meals | 1079 (14.33%) | 192 (8.38%) | 182 (9.73%) | 150 (8.27%) | 555 (35.69%) |
| Bills and banking | 1139 (84.88%) | 223 (9.73%) | 200 (10.69%) | 134 (7.39%) | 582 (37.43%) |
| **Self-care activities** |  |  |  |  |  |
| Eating | 316 (4.20%) | 50 (2.18%) | 52 (2.78%) | 24 (1.32%) | 190 (12.22%) |
| Dressing | 632 (8.39%) | 90 (3.93%) | 128 (6.84%) | 82 (4.52%) | 332 (21.35%) |
| Bathing | 387 (5.14%) | 66 (2.88%) | 68 (3.63%) | 36 (1.98%) | 217 (13.95%) |
| Toileting | 221 (2.93%) | 35 (1.53%) | 33 (1.76%) | 14 (0.77%) | 139 (8.94%) |
| **Mobility activities** |  |  |  |  |  |
| Indoor mobility | 492 (6.53%) | 71 (3.10%) | 90 (4.81%) | 50 (2.76%) | 281 (18.07%) |
| Outdoor mobility | 703 (9.33%) | 113 (4.93%) | 147 (7.86%) | 103 (5.68%) | 340 (21.86%) |
| Transferring from bed | 361 (4.79%) | 53 (2.31%) | 59 (3.15%) | 31 (1.71%) | 218 (14.02%) |

Note: Some-Somatic refers to the some-somatic with moderate cognitive impairment group; dementia includes possible and probable dementia classifications assessed in NHATS
